# Risk factors for severe acute respiratory coronavirus virus 2 (SARS-CoV-2) seropositivity among nursing home staff

**DOI:** 10.1017/ash.2021.193

**Published:** 2021-10-28

**Authors:** Avnika B. Amin, Joseph T. Kellogg, Carly Adams, William C. Dube, Matthew H. Collins, Benjamin A. Lopman, Theodore M. Johnson, Joshua Weitz, Scott K. Fridkin

**Affiliations:** 1 Department of Epidemiology, Emory University Rollins School of Public Health, Atlanta, Georgia; 2 Division of Infectious Diseases, Department of Medicine, Emory University School of Medicine, Atlanta, Georgia; 3 The Hope Clinic of the Emory Vaccine Center, Division of Infectious Diseases, Emory University School of Medicine, Atlanta, Georgia; 4 Department of Environmental Health, Emory University Rollins School of Public Health, Atlanta, Georgia; 5 Division of General Internal Medicine, Department of Medicine, Emory University School of Medicine, Atlanta, Georgia; 6 Department of Family and Preventive Medicine, Emory University School of Medicine, Atlanta, Georgia; 7 School of Biological Sciences, Georgia Institute of Technology, Atlanta, Georgia; 8 School of Physics, Georgia Institute of Technology, Atlanta, Georgia

## Abstract

**Objectives:**

To estimate prior severe acute respiratory coronavirus virus 2 (SARS-CoV-2) infection among skilled nursing facility (SNF) staff in the state of Georgia and to identify risk factors for seropositivity as of fall 2020.

**Design:**

Baseline survey and seroprevalence of the ongoing longitudinal Coronavirus 2019 (COVID-19) Prevention in Nursing Homes study.

**Setting:**

The study included 14 SNFs in the state of Georgia.

**Participants:**

In total, 792 SNF staff employed or contracted with participating SNFs were included in this study. The analysis included 749 participants with SARS-CoV-2 serostatus results who provided age, sex, and complete survey information.

**Methods:**

We estimated unadjusted odds ratios (ORs) and 95% confidence intervals (95% CIs) for potential risk factors and SARS-CoV-2 serostatus. We estimated adjusted ORs using a logistic regression model including age, sex, community case rate, SNF resident infection rate, working at other facilities, and job role.

**Results:**

Staff working in high-infection SNFs were twice as likely (unadjusted OR, 2.08; 95% CI, 1.45–3.00) to be seropositive as those in low-infection SNFs. Certified nursing assistants and nurses were 3 times more likely to be seropositive than administrative, pharmacy, or nonresident care staff: unadjusted OR, 2.93 (95% CI, 1.58–5.78) and unadjusted OR, 3.08 (95% CI, 1.66–6.07). Logistic regression yielded similar adjusted ORs.

**Conclusions:**

Working at high-infection SNFs was a risk factor for SARS-CoV-2 seropositivity. Even after accounting for resident infections, certified nursing assistants and nurses had a 3-fold higher risk of SARS-CoV-2 seropositivity than nonclinical staff. This knowledge can guide prioritized implementation of safer ways for caregivers to provide necessary care to SNF residents.

Skilled nursing facilities (SNFs), commonly referred to as nursing homes, are settings at high risk for outbreaks of respiratory diseases like coronavirus 2019 (COVID-19).^1–3^ SNF residents are at extremely high risk for severe acute respiratory coronavirus virus 2 (SARS-CoV-2) transmission from close contact with staff during activities of daily living (ADL) assistance, rehabilitative care, and respiratory care.^1,4^ Additionally, residents suffer from high morbidity and mortality related to COVID-19 because they tend to be older and have more chronic medical conditions than the general population.^1,4^

To limit the introduction and transmission of SARS-CoV-2 in SNFs and protect vulnerable residents, on March 13, 2020, the Centers for Medicare and Medicaid Services (CMS) recommended restricting resident visitation outside of end-of-life situations.^5^ Nevertheless, outbreaks continued to occur in SNFs, some of which may have resulted from inadvertent SARS-CoV-2 transmission via nursing home staff.^6^ Both residents and staff may have transmitted and acquired SARS-CoV-2 from each other. SNFs may have amplified SARS-CoV-2 transmission between May 25 and November 22, 2020; the 572,135 COVID-19 cases reported by SNFs during this period accounted for a large proportion of total infections.^7^

Considering that SNF staff may both initiate SNF transmission chains and also acquire infections at SNFs, identifying risk factors for SNF staff infection would support targeted infection control and prevention efforts. Currently, evidence for risk factors is scarce. Job role may be important, with some evidence of increased risk for nursing assistants and social workers.^8^ Resident-care SNF staff have different types and intensities of contact with residents (eg, ADL care) compared to hospital staff contacts with patients. Previous work identifying risk factors for hospital staff (eg, community contacts and spread,^9,10^ contact with COVID-19 patients,^11–13^ and support staff roles)^14,15^ may not apply to SNF staff. Risks to nonresident care SNF staff are also uncertain. With widespread SARS-CoV-2 transmission, reliance on self-reported symptomatic disease is insufficient for evaluation of specific risk factors; two-thirds of asymptomatic SNF staff may be SARS-CoV-2 seropositive after outbreaks in SNFs where they work.^16^ Our objectives were to estimate prior SARS-CoV-2 infection among SNF staff at 14 nursing homes in the state of Georgia and to identify risk factors for seropositivity as of fall 2020.

## Methods

We recruited participants from 14 SNFs selected from 54 facilities affiliated with 4 healthcare systems in Georgia (Supplemental Fig. 1 online). All facilities provided postacute and long-term care services. No facilities cared for ventilator-dependent residents. All facilities followed CMS-required visitor restrictions for the duration of recruitment and data collection, which were part of the ongoing longitudinal COVID-19 Prevention in Nursing Homes (COPING) study. Participants were recruited from August 25 to November 22, 2020 for the first study visit, which was the focus of analysis. This study was evaluated and approved by the Emory University IRB (#00000900). All participants provided consent.

### Study population

Eligible SNF staff were ≥18 years old and were employed or contracted by participating facilities. Recruitment included e-mails, flyers, and conversations between nursing leadership and staff departments. Study staff visited each facility during shift changes over a 2- or 3-day period to recruit, consent, collect specimens, and administer surveys.

### Serologic testing and survey data collection

The SNF staff first completed an electronic survey regarding contacts with confirmed or suspected COVID-19 cases (within and outside work), COVID-19 symptom history, workplace masking and social distancing, and occupational activities (primary job role, shifts worked each month, resident care involvement, and facilities worked at). After completing the survey and under study staff supervision, SNF staff used lancets to self-collect dried blood spot samples for SARS-CoV-2 serology testing. Staff provided sex, age, and their residential ZIP code upon sample collection. Race and ethnicity were not available for this analysis. Sample cards air dried for 15 minutes before packaging, storage, and shipment to the testing laboratory (Molecular Testing Laboratories, Vancouver, WA) within 2 days of acquisition. A qualitative enzyme-linked immunosorbent assay (EUROIMMUN, Mountain Lakes, NJ) was used to test samples for the presence of anti–SARS-CoV-2 spike protein immunoglobulin G.

### External data sources

To assess community-based exposure to SARS-CoV-2, we used confirmed COVID-19 case data from the Georgia Department of Public Health. The number of documented COVID-19 cases occurring in a participant’s residential ZIP code tabulation area (ZCTA) from March 1, 2020, to 2 weeks before the serology test was used to approximate staff’s cumulative community exposure to SARS-CoV-2 (termed community case rates). ZCTA-specific population estimates were obtained from the 2019 American Community Survey^17^ to provide a population denominator. To assess facility-based exposure to SARS-CoV-2, we used data on confirmed SNF resident COVID-19 cases reported to CMS.^18^ The documented number of total resident cases from the first week of reporting and new cases from the first week of reporting until 2 weeks before the serology test was used to approximate facility infection rates. The first week of reporting was either May 24 or May 31, 2020, for each participating facility. Facility-specific bed sizes from CMS data provided a denominator to account for differences in facility size and to calculate facility resident COVID-19 cases per 100 beds. For both potential SARS-CoV-2 exposures, the 2-week window before serology testing accounted for the typical minimum lag between infection and seroconversion.^19^

### Statistical analysis

Participant seropositivity by facility and frequency of COVID-19 symptoms experienced by seropositive participants were calculated. To examine risk factors for staff seropositivity, we a priori identified age, sex, job role, community exposure (known contact with community cases and cumulative community infection rates), facility case rates [dichotomized into high-burden (ie, >15 cases per 100 beds) and low-burden among SNF residents based on the approximate sample median], known contact with cases in facilities, and working at multiple facilities as risk factors to consider. Age was categorized into 4 groups: <40, 40–49, 50–59, and ≥60 years. Staff responses were mapped to 6 job categories to compare types of contact with residents: (1) healthcare administration, pharmacy, and other nonresident care; (2) resident activities, environmental services, and food services; (3) social work and physical, occupational, respiratory, and speech therapy; (4) certified nursing assistants; (5) nurses (including registered nurses and licensed-practical nurses); and (6) physicians and advanced practice providers. We estimated unadjusted odds ratios (ORs) and 95% confidence interval (95% CIs) for these and other potential risk factors and SARS-CoV-2 serostatus.

We used logistic regression to estimate adjusted ORs for seropositivity, including all a priori identified variables, shifts worked monthly (≤10 or 10+), proportion of work time directly caring for residents (≤50% or >50%), and a facility-level random intercept (fully adjusted model). We could not examine interactions between job role and facility resident COVID-19 burden in the model due to insufficient sample size. We determined that age, sex, job role, community cases, and facility resident cases were established risk factors to be retained in the model. The other variables were assessed for potential removal and retained if >10% change in odds ratios (ORs) from the fully adjusted ORs occurred.

## Results

Of 2,053 eligible SNF staff, we enrolled 792 staff (38.6%) and included 749 in this analysis; 23.5% were SARS-CoV-2 seropositive. Seropositivity by facility varied from 5.8% to 48.0% (Table 1). Only 47 (26.7%) of 176 seropositive SNF staff reported at least 1 respiratory symptom (cough, shortness of breath, or difficulty breathing) in the 3 months before serology sample collection (Supplementary Table 1 online). Cumulative facility resident infection rates ranged from 0.4 to 106.7 infections per 100 beds (Supplementary Fig. 2A online). Cumulative community case rates ranged from 0.94 to 13.10 cases per 100 ZCTA population (Supplementary Fig. 2B online).

**Table 1.** Skilled Nursing Facility Bed Size, Staff Enrollment, Inclusion in Analysis, and SARS-CoV-2 Seropositivity, by Facility September–October, 2020.

Facility	Beds	>15 Resident Infections per 100 Beds^a^	Enrolled^b^		Included in Analysis^c^		SARS-CoV-2 seropositive^d^	
	No.		No.	(%)	No.	(%)	No.	(%)
Facility 1	125	No	55	(45.8)	52	(94.5)	3	(5.8)
Facility 2	150	Yes	85	(39.4)	76	(89.4)	7	(9.2)
Facility 3	236	No	100	(33.3)	99	(99.0)	11	(11.1)
Facility 4	138	No	70	(42.2)	68	(97.1)	11	(16.2)
Facility 5	125	Yes	22	(22.4)	22	(100.0)	4	(18.2)
Facility 6	76	No	32	(42.1)	31	(96.9)	6	(19.4)
Facility 7	165	Yes	61	(42.4)	58	(95.1)	13	(22.4)
Facility 8	163	Yes	58	(58.0)	57	(98.3)	14	(24.6)
Facility 9	250	No	83	(54.2)	74	(89.2)	22	(29.7)
Facility 10	117	Yes	33	(53.2)	33	(100.0)	11	(33.3)
Facility 11	137	Yes	44	(25.7)	43	(97.7)	16	(37.2)
Facility 12	152	Yes	67	(54.0)	61	(91.0)	23	(37.7)
Facility 13	100	Yes	57	(44.5)	50	(87.7)	23	(46.0)
Facility 14	119	Yes	25	(25.0)	25	(100.0)	12	(48.0)

a Based on cumulative resident COVID-19 cases per 100 beds occurring no more than two weeks before serology collection. Because serology collection occurred on multiple days, slight changes in the cumulative case rates occurred. No facility case rates changed from ≤15 to >15 cases per 100 beds.

b Consented participants who provided ablood sample, and percent of total healthcare personnel.

c Enrolled participants not missingage, residential zip code, or answers to survey questions used in analysis.

d Participants included in analysis who tested positive for SARS-CoV-2 antibodies.

Unadjusted odds ratios for SARS-CoV-2 seropositivity (Table 2) indicated that staff who spent >50% of their work time on direct resident care were 57% more likely (OR, 1.57; 95% CI, 1.12–2.21) to be seropositive than those who spent ≤50% of their work time on direct care. Staff working in high-infection facilities were approximately twice as likely (OR, 2.08; 95% CI, 1.45–3.00) to be seropositive as those working in low-infection facilities. However, community COVID-19 infection rates were not associated with higher odds of being seropositive. Self-reported compliance with infection prevention practice (distancing at work, universal masking) and known workplace COVID-19 contact were not associated with seropositivity.

**Table 2.** Healthcare Personnel Characteristics by Serostatus, and Unadjusted Odds Ratios for Potential Risk Factors, September–October 2020

	Overall (n = 749)		Seronegative (n = 573)		Seropositive (n = 176)		Unadjusted odds ratios and 95% CIs	
	No.	(%)	No.	(%)	No.	(%)	OR	95% CI
**Sex**								
Female	631	(84.2)	479	(84)	152	(86.4)	1.00	Ref
Male	118	(15.8)	94	(16)	24	(13.6)	0.81	(0.49–1.29)
**Age, y**								
<40	203	(27.1)	161	(28.1)	42	(23.9)	1.00	Ref
40–49	193	(25.8)	149	(26.0)	44	(25.0)	1.13	(0.70–1.83)
50–59	226	(30.2)	170	(29.7)	56	(31.8)	1.26	(0.80–2.00)
60+	127	(17.0)	93	(16.2)	34	(19.3)	1.40	(0.83–2.36)
**Job role**								
No direct resident care^a^	111	(14.8)	97	(16.9)	14	(8.0)	1.00	Ref
Activities, environmental or food services	153	(20.4)	128	(22.3)	25	(14.2)	1.35	(0.67–2.80)
Physician or advanced practice provider	22	(2.9)	20	(3.5)	2	(1.1)	0.73	(0.10–2.97)
Social work, physical, occupational, respiratory, or speech therapy	89	(11.9)	68	(11.9)	21	(11.9)	2.12	(1.01–4.58)
Certified nursing assistant	187	(25.0)	131	(22.9)	56	(31.8)	2.93	(1.58–5.78)
Nurse	187	(25.0)	129	(22.5)	58	(33.0)	3.08	(1.66–6.07)
**Work at other facilities**								
*No or prefer not to answer*	670	(89.5)	516	(90.1)	154	(87.5)	1.00	Ref
*Yes*	79	(10.5)	57	(9.9)	22	(12.5)	1.30	(0.75–2.17)
**No. of shifts in prior month**								
≤10	318	(42.5)	251	(43.8)	67	(38.1)	1.00	Ref
>10	431	(57.5)	322	(56.2)	109	(61.9)	1.27	(0.90–1.80)
**Proportion of work directly caring for residents**								
≤50%	405	(54.1)	325	(56.7)	80	(45.5)	1.00	Ref
>50%	344	(45.9)	248	(43.3)	96	(54.5)	1.57	(1.12–2.21)
**Known COVID-19 contact at work^b^**								
No or unknown	432	(57.7)	334	(58.3)	98	(55.7)	1.00	Ref
Yes	317	(42.3)	239	(41.7)	78	(44.3)	1.11	(0.79–1.56)
**Known COVID-19 contact outside of work**								
No or unknown	693	(92.5)	527	(92.0)	166	(94.3)	1.00	Ref
Yes	56	(7.5)	46	(8.0)	10	(5.7)	0.70	(0.32–1.36)
**Universal masking at work**								
≥80% of the time	640	(85.4)	482	(84.1)	158	(89.8)	1.00	Ref
<80% of the time	109	(14.6)	91	(15.9)	18	(10.2)	0.61	(0.34–1.02)
**Consistently distance at work**								
No or unsure	152	(20.3)	120	(20.9)	32	(18.2)	1.00	Ref
Yes	597	(79.7)	453	(79.1)	144	(81.8)	1.19	(0.78–1.86)
**Cumulative facility resident COVID-19 infections**								
Low (≤15 cases per 100 beds)	324	(43.3)	271	(47.3)	53	(30.1)	1.00	Ref
High (>15 cases per 100 beds)	425	(56.7)	302	(52.7)	123	(69.9)	2.08	(1.45–3.00)
Cumulative COVID-19 cases per 100 in HCP residential ZIP code tabulation area^c^	2.8 (2.3–3.3)		2.8 (2.3–3.2)		2.9 (2.5–3.5)		1.11	(0.93–1.33)

Note. CI, confidence interval; OR, odds ratio; HCP, healthcare personnel.

a Healthcare administration, pharmacy, or nonresident care.

b Contacts at work included residents or staff.

c Presented as median (IQR) instead of No. (%)

In multivariate analyses, working at multiple facilities, age, sex, and community case rates were not associated with odds of seropositivity (Table 3). Job role and working at a high-infection facility were significantly associated with odds of seropositivity (Table 3). When other model variables were controlled for, certified nursing assistants and nurses were >3 times as likely to be seropositive than administrative, pharmacy, or nonresident care staff: OR, 3.43 (95% CI, 1.74–6.76) and OR, 3.15 (95% CI, 1.61–6.14) (Supplementary Fig. 3 online and Table 3). As in univariate analysis, staff at high-infection facilities had significantly elevated odds of seropositivity (OR, 2.30; 95% CI, 1.09–4.82). Although working at multiple facilities was not a statistically significant risk factor, it was retained because its removal resulted in a >10% change in odds ratios for the other variables in the model. Proportion of work time spent on direct resident care was no longer associated with seropositivity once job category was included in the regression model, suggesting that the nature of resident care is more related to seropositivity than time at bedside alone.

**Table 3.** Final Model Results With Age, Sex, Job Role, Cumulative COVID-19 Incidence per 100 Population, Cumulative Facility Resident COVID-19 Burden, and Whether or Not Participants Worked at Other Facilities

Category	Odds Ratio	(95% Cl)
**Age, y**		
<40	1.00	Ref
40-49	1.23	(0.74–2.04)
50-59	1.36	(0.83–2.21)
60+	1.61	(0.92–2.82)
**Sex**		
Female	1.00	Ref
Male	1.10	(0.65–1.86)
**Working at other facilities**		
No or prefer not to answer	1.00	Ref
Yes	1.31	(0.73–2.34)
Cumulative community COVID-19 incidence per 100 population	1.03	(0.82–1.30)
**Cumulative facility resident infections**		
Low (≤15 cases per 100 beds)	1.00	Ref
High (>15 cases per 100 beds)	2.30	(1.09–4.82)
**Job role**		
Healthcare administration, pharmacy, or nonpatient care	1.00	Ref
Activities, environmental services, or food services	1.62	(0.78–3.37)
Certified nursing assistant	3.43	(1.74–6.76)
Nurse	3.15	(1.61–6.14)
Physician or advanced practice provider	0.70	(0.14–3.61)
Social work or physical, occupational, respiratory, or speech therapy	2.13	(0.98–4.64)

Note. CI, confidence interval; Ref, reference.

## Discussion

In this study, the SARS-CoV-2 seropositivity rate of 23.5% among SNF staff was higher than the estimated 14% seropositivity in the US population at the time of data collection completion,^20^ which highlights the disproportionate impact of the pandemic on SNF staff.^8^ Working at high-infection facilities was a risk factor for SARS-CoV-2 seropositivity. Even after accounting for resident infections at SNFs, certified nursing assistants and nurses appear to have an ˜3-fold higher risk of SARS-CoV-2 seropositivity compared to administrative, pharmacy, or nonresident-care staff. Time spent on direct care may not affect seropositivity risk once job type is considered, suggesting that distinct types of resident care or staff-to-staff interactions inherent to different job roles may put staff at increased risk relative to each other. Interestingly, working at multiple facilities did not appear to be a risk factor for seropositivity, even though other recent work has estimated SNFs share worker connections with an average of 7 other facilities.^6^ However, SNF staff may be reluctant to report working at other facilities, a limitation of this analysis. Furthermore, our study did not find evidence that the community exposure rate was an important risk factor for infection, in contrast to prior evidence relating community prevalence to risk of infection for acute-care staff.^9,10^

Our findings of heightened risk for certified nursing and nurses are supported by similar findings from a recent serosurvey of 1,500 SNF staff in Rhode Island.^8^ However, community contacts were not associated with seropositivity. The discrepancy could reflect recall bias, breakdown in effective testing and contact tracing, or better infection prevention awareness in the general community. We also observed twice the seroprevalence among SNF staff (24% vs 13% in the earlier Rhode Island study). Although smaller, our study captured a variety of geographic and temporal diversity in community and facility infections.

This study had several limitations. We used self-reported data on exposures, which introduces possible recall error into our risk factor assessment, and we do not have data on race or ethnicity. Duties for the same job title may vary by facility, and we cannot ascertain the specific activities of participants who reported working in multiple facilities, although only 11% of SNF staff reported such work. We also cannot determine where SNF staff acquired infection (ie, in the community or at work). Asymptomatic and presymtomatic transmission may facilitate unrecognized transmission and contacts in both the community and the workplace; individuals may be less vigilant with infection prevention measures in the absence of respiratory symptoms. Regardless, it seems clear that early in this pandemic, SNFs provide conditions to amplify SARS-CoV-2 transmission beyond what is observed in the community. In this study, we addressed the lack of evidence for risk factors for SNF staff SARS-CoV-2 seropositivity, and our findings provide insight into differences between acute-care and long-term care risk factors.

Overall, US-based SNF staff and residents have been severely affected by the COVID-19 pandemic. Our study helps define and quantify the risks to SNF staff. The consistency between our work and the earlier Rhode Island study suggests that the work activities of certified nursing assistants and nurses put these personnel at higher risk for SARS-CoV-2 infection early in the pandemic. We did not find community infections to be a significant risk factor; however, we are limited in capturing heterogeneity in individual-level community-derived risk. Given their elevated risks for infection, certified nursing assistants and nurses may have needed better implementation of recommended infection prevention measures in their existing workflows given the types of interactions required to serve SNF residents and interact with other staff (eg, close physical proximity, small spaces, prolonged contact times). Further study is urgently needed to determine and implement safer ways to provide necessary care to senior populations in SNFs and avoid transmission of respiratory viruses.

## Supplementary material

For supplementary material accompanying this paper visit https://doi.org/10.1017/ash.2021.193. click here to view supplementary material
