# Supplementary material for: Risk factors for severe acute respiratory coronavirus virus 2 (SARS-CoV-2) seropositivity among nursing home staff
Source: Antimicrob Steward Healthc Epidemiol. 2021 Oct 28;1(1):e35. doi: 10.1017/ash.2021.193 (PMC9495639; doi:10.1017/ash.2021.193)
Supplement: Supplementary file 1 [file S2732494X21001935sup001.docx]

**Appendix**

| 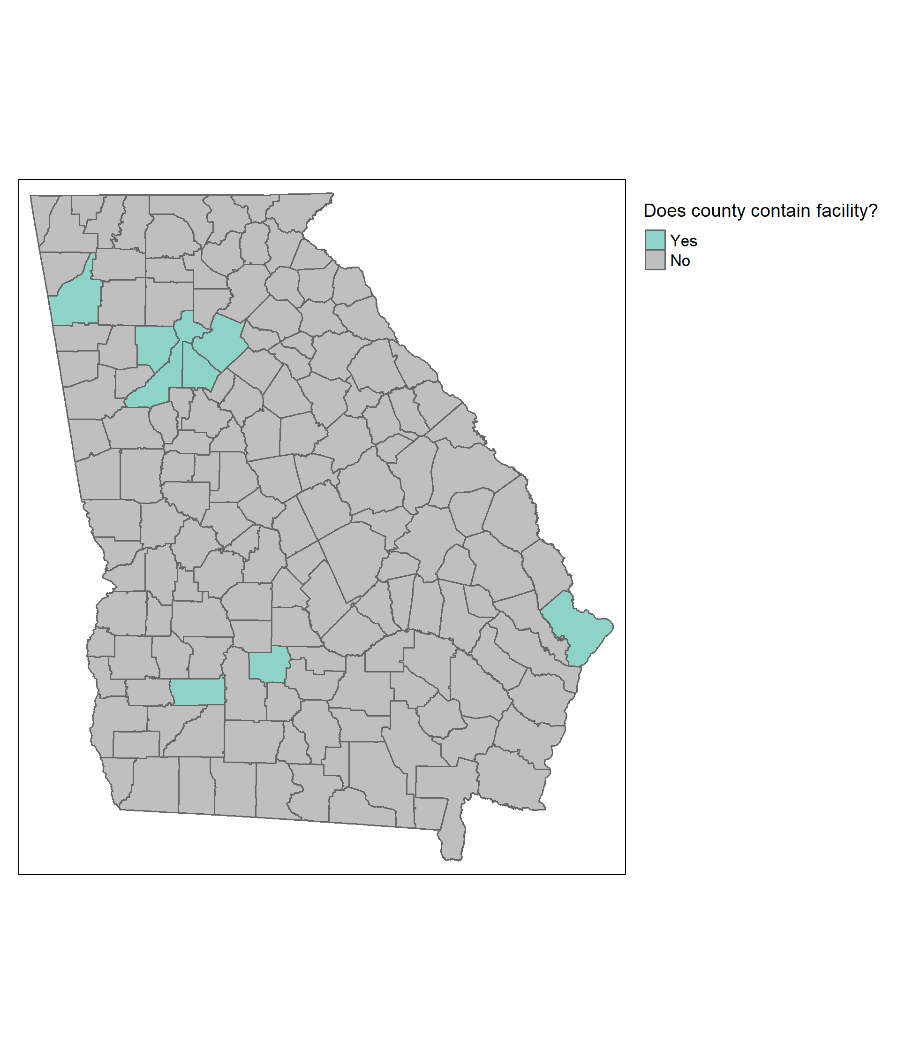 |
| --- |
| **Supplemental Figure 1. Locations of skilled nursing facilities in Georgia involved in the study.** Highlighted counties indicate the location of at least one participating facility. |

| **Supplemental Table 1. Symptoms experienced by SARS-CoV-2 seropositive healthcare providers in the three months prior to sample collection.** | | |
| --- | --- | --- |
|  |  |  |
|  | ***N*** | ***(%)*** |
| Cough, shortness of breath, or difficulty breathing (at least one) |  |  |
| No or unsure | 129 | (73.3) |
| Yes | 47 | (26.7) |
| Fever, chills, rigors, myalgia, headache, sore throat, or altered sense of taste/smell (at least two) |  |  |
| No or unsure | 131 | (74.4) |
| Yes | 45 | (25.6) |
| Severe respiratory illness with pneumonia and/or acute respiratory distress syndrome |  |  |
| No or unsure | 167 | (94.9) |
| Yes | 9 | (5.1) |
| At least one of the above symptoms |  |  |
| No or unsure | 117 | (66.5) |
| Yes | 59 | (33.5) |

| **A** | 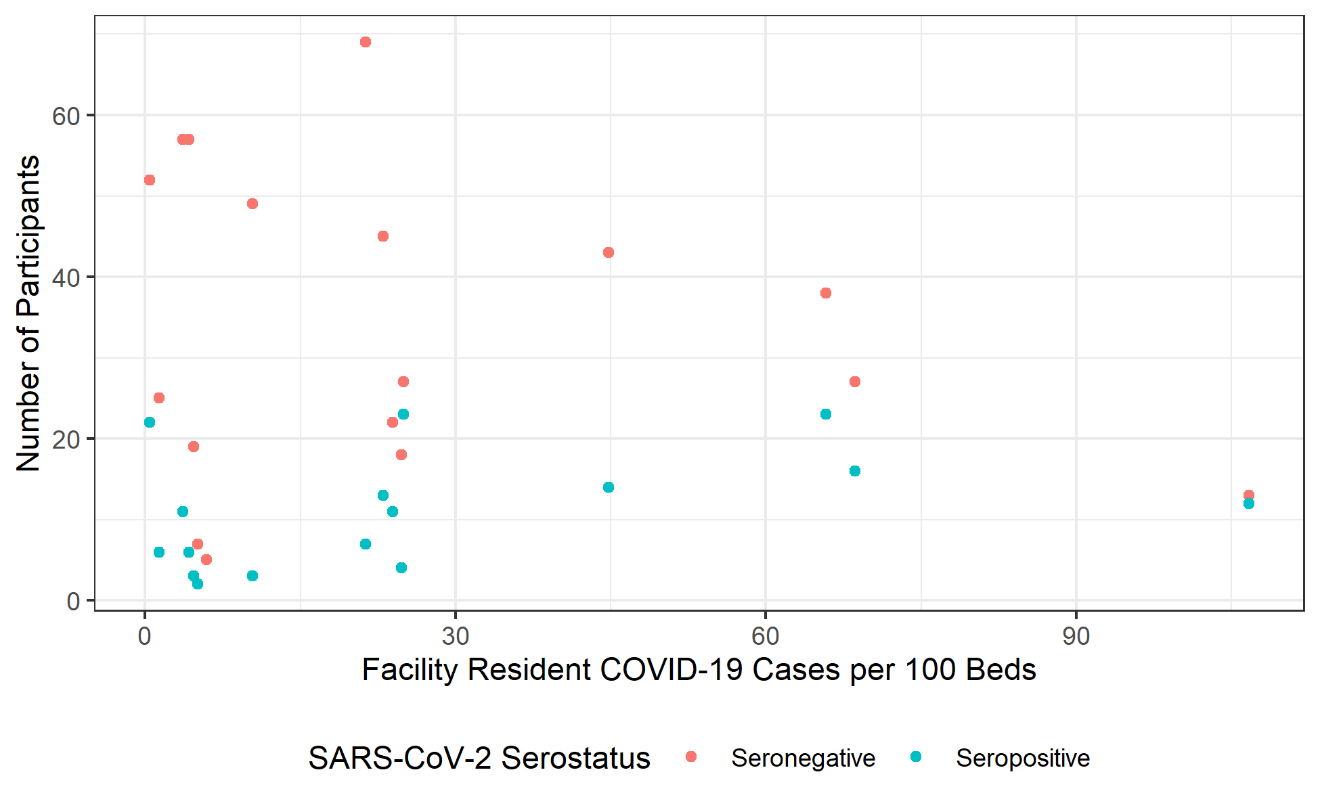 |
| --- | --- |
| **B** | 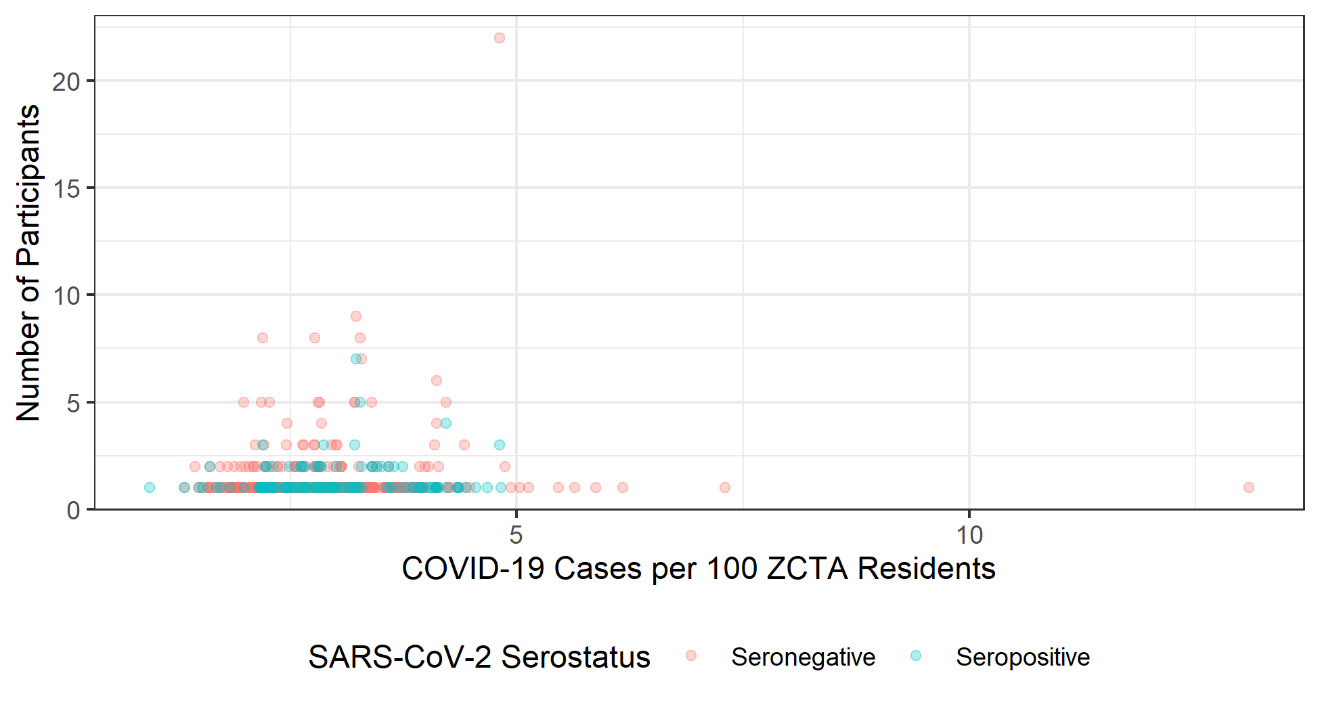 |
| **Supplemental Figure 2. (A) Distribution the facility’s resident case rate (COVID-19 cases per 100 beds) by healthcare personnel (HCP) serostatus, and (B) distribution of HCP serostatus by community case rate (cumulative COVID-19 cases per 100 in zip code tabulation area [ZCTA]) by HCP serostatus.** | |

| 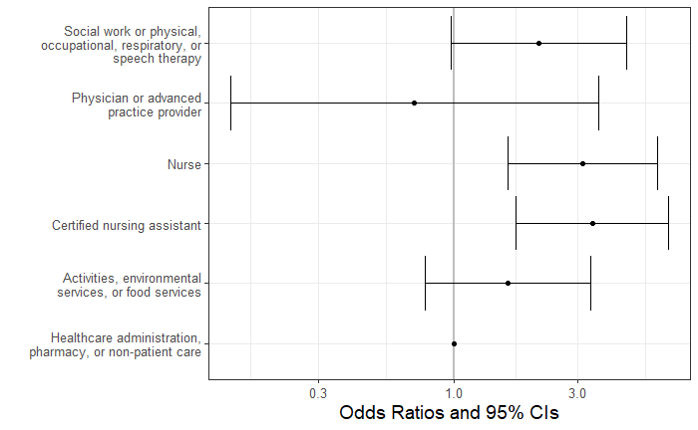 |
| --- |
| **Supplemental Figure 3. Adjusted odds ratios and 95% confidence intervals (CI) for the reported job role on seropositivity for SARS Cov-2 antibody accounting for age, sex, cumulative COVID-19 incidence per 100 residents in the community, cumulative facility resident COVID-19 burden, and whether or not participants worked at other facilities.** |
